# Dietary Supplementation of Astaxanthin Improved the Growth Performance, Antioxidant Ability and Immune Response of Juvenile Largemouth Bass (*Micropterus salmoides*) Fed High-Fat Diet

**DOI:** 10.3390/md18120642

**Published:** 2020-12-15

**Authors:** Shiwei Xie, Peng Yin, Lixia Tian, Yingying Yu, Yongjian Liu, Jin Niu

**Affiliations:** 1 Guangdong Provincial Key Laboratory of Improved Variety Reproduction in Aquatic Economic Animals, Institute of Aquatic Economic Animals, School of Life Sciences, Sun Yat-Sen University, Guangzhou 510275, China; xiesw@gdou.edu.cn (S.X.); peng.yin@hi.no (P.Y.); lsstlx@mail.sysu.edu.cn (L.T.); edls@mail.sysu.edu.cn (Y.L.); 2 Laboratory of Aquatic Animal Nutrition and Feed, Fisheries College, Guangdong Ocean University, Zhanjiang 524088, China; 3 Institute of Marine Research (IMR), NO-5817 Bergen, Norway; 4 Guangdong Key Laboratory of Animal Molecular Design and Precision Breeding, School of Life Science and Engineering, Foshan University, Foshan 528527, China; yuyin123@fosu.edu.cn

**Keywords:** apoptosis, inflammation, lipid metabolism, anti-oxidant, high-fat diet

## Abstract

High-fat diet (HFD) usually induces oxidative stress and astaxanthin is regarded as an excellent anti-oxidant. An 8-week feeding trial was conducted to investigate the effects of dietary astaxanthin supplementation on growth performance, lipid metabolism, antioxidant ability, and immune response of juvenile largemouth bass (*Micropterus salmoides*) fed HFD. Four diets were formulated: the control diet (10.87% lipid, C), high-fat diet (18.08% lipid, HF), and HF diet supplemented with 75 and 150 mg kg^−1^ astaxanthin (HFA1 and HFA2, respectively). Dietary supplementation of astaxanthin improved the growth of fish fed HFD, also decreased hepatosomatic index and intraperitoneal fat ratio of fish fed HFD, while having no effect on body fat. Malondialdehyde content and superoxide dismutase activity were increased in fish fed HFD, astaxanthin supplementation in HFD decreased the oxidative stress of fish. The supplementation of astaxanthin in HFD also reduced the mRNA levels of *Caspase 3*, *Caspase 9*, *BAD*, and *IL15*. These results suggested that dietary astaxanthin supplementation in HFD improved the growth performance, antioxidant ability and immune response of largemouth bass.

## 1. Introduction

The largemouth bass, *Micropterus salmoides*, is one of the most important economical freshwater fish in China, and its production in 2019 reached more than 470,000 tons [1]. Recent research revealed that largemouth bass has a limited ability to utilize starch, the starch content in the feed usually below 10% [2,3], so dietary lipids serve as the main provider of energy in the diet.

Besides the energy, dietary lipid provides other important substances such as essential fatty acids, phospholipids and sterols for maintaining cell normal structure and biological function [4]. Due to the protein-sparing effect of dietary lipids in aquatic feeds, high-fat diet (HFD) is widely used to save costs and reduce nitrogen waste in aquaculture recently [5,6,7]. However, excessive dietary lipid may lead to abnormal lipid accumulation in liver, abdominal adipose tissues and muscle. In many studies, long-term intake of HFD induced reactive oxygen species (ROS) production, oxidative stress and inflammation to fish, and finally negatively affected the growth performance and health of fish [8,9,10]. Previous research indicated that lipid accumulation of largemouth bass increased with the increasing dietary lipid levels [11], and several studies revealed that high dietary lipid (18–20%) impaired the growth and health of largemouth bass [12,13].

Astaxanthin is a potent lipid-soluble marine keto-carotenoid with auspicious effects on human and animal health [14]. It is found widely in aquatic animals and some other organisms, but its de novo synthesis is limited to several bacteria, protists, fungi, algae and plants, and synthetic astaxanthin accounts for >95% of the world market currently [15]. Astaxanthin is an important colorant in the crustacean and salmonid feed industry [16,17], and is also an additive with auspicious effects on egg quality [18]. More importantly, astaxanthin acts as a safeguard against oxidative stress through different mechanisms such as scavenging of radicals and neutralizing of singlet oxygen, and astaxanthin also plays a critical role in anti-inflammatory response through modulating the cytokines production, NF-κB signaling pathway and apoptotic pathways [14,15,19,20]. Dietary supplementation of astaxanthin has been shown to improve the growth performance, anti-oxidative capacity and immune response of shrimp, crab and a variety of fish [16,21,22,23,24]. Astaxanthin has been reported to alleviate the oxidative stress of rats induced by HFD [25,26], while there is no research evaluating the effect of dietary astaxanthin supplementation on fish fed HFD.

The aim of this study was to evaluate the effects of HFD and astaxanthin supplementation on growth performance, lipid metabolism, anti-oxidative ability and immune response of juvenile largemouth bass.

## 2. Results

### 2.1. Growth Performance, Feed Utilization and Somatic Parameters

Dietary astaxanthin supplementation on growth performance, feed utilization and somatic parameters of juvenile largemouth bass fed HFD are shown in Table 1. Results showed that there were no differences in final body weight (FBW), weight gain rate (WG) and specific growth rate (SGR) among fish fed diet C and HF. Dietary supplementation of 75 mg kg^−1^ astaxanthin in HF diet significantly increased FBW, WG and SGR of fish (*p* < 0.05). Protein efficiency ratio (PER) of fish fed diet C and HF were significantly lower compared with those fed diets supplemented with 75 and 150 mg kg^−1^ astaxanthin (*p* < 0.05). Viscerosomatic index (VSI) was lower in fish fed the control diet compared to those fed other diets (*p* < 0.05). Hepatosomatic index (HSI) and intraperitoneal fat ratio (IPF) were significantly higher in fish fed HF diet compared to those fed control diet. Compared to fish fed HFD, dietary supplementation of 75–150 mg kg^−1^ astaxanthin decreased the HSI of fish (*p* < 0.05), and dietary supplementation of 150 mg kg^−1^ astaxanthin decreased the IPF of fish (*p* < 0.05).

### 2.2. Whole Body and Muscle Proximate Composition

The moisture content in whole-body and ash content in muscle was significantly higher in fish fed diet C than those fed other diets (*p* < 0.05) (Table 2). In contrast, the lipid content in the whole body and muscle was lower in fish fed diet C compared to those fed the other three diets (*p* < 0.05). The protein contents of the whole body showed no differences among the four treatments (*p* > 0.05). Likewise, no differences were found in moisture and crude protein contents of muscle (*p* > 0.05).

### 2.3. Plasma and Hepatic Biochemical Indexes

The results of biochemical parameters in plasma and liver are presented in Table 3. Triglyceride (TG) and total cholesterol (TC) in plasma were significantly higher in fish fed diet HF than those fed the control diet (*p* < 0.05). Dietary supplementation of 75 and 150 mg kg^−1^ astaxanthin significantly decreased the TG content in plasma (*p* < 0.05). Fish fed diet HFA2 obtained a higher HDL/LDL ratio compared to those fed diets HF and HFA1. Dietary lipid levels and astaxanthin had no effect on LDH activity in the plasma.

Fish fed diet HFA1 showed higher TG content in the liver compared to those fed diet C (*p* < 0.05). Fish fed diet HF showed higher TC content in the liver than those fed diet C and HFA1 (*p* < 0.05). The lipase activity in the liver was similar among fish fed the four diets (*p* > 0.05).

### 2.4. Lipid Accumulation in the Liver

**Figure 1:**
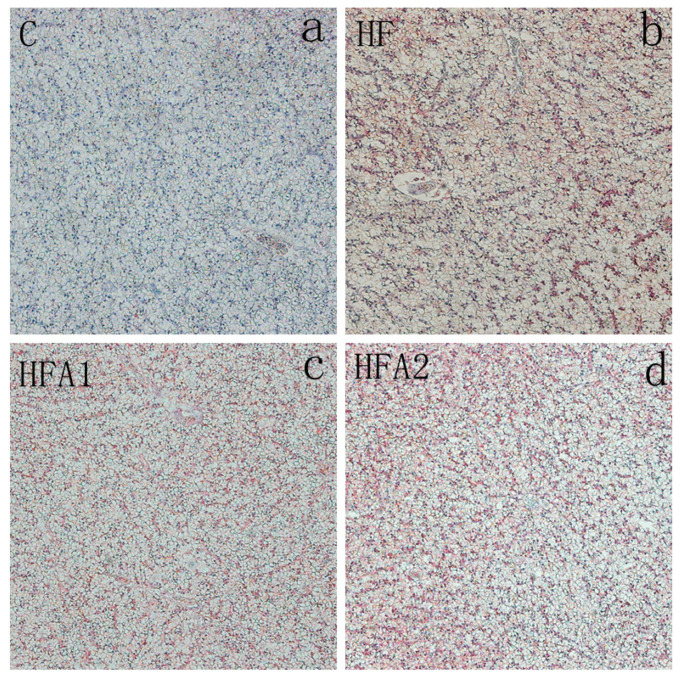
Effect of high-fat diet and astaxanthin on hepatic lipid accumulation of juvenile largemouth bass. Lipid droplets and nuclei are dyed in red and blue by oil red O staining, respectively (100 × magnification). (**a**), the lipid accumulation of juvenile largemouth bass fed control diet; (**b**), the lipid accumulation of juvenile largemouth bass fed high fat diet (HF); (**c**), the lipid accumulation of juvenile largemouth bass fed HFA1; (**d**), the lipid accumulation of juvenile largemouth bass fed HFA2.

To evaluate the lipid accumulation in the liver, oil red O staining was performed and shown in Figure 1. Lipid droplets and nuclei are dyed in red and blue, respectively. In this study, there are fewer lipid droplets in fish fed diet C compared to fish fed other diets. Dietary supplementation of astaxanthin had no effect on hepatic lipid accumulation of largemouth bass.

### 2.5. Lipid Metabolism Related Gene Expression in Liver

**Figure 2:**
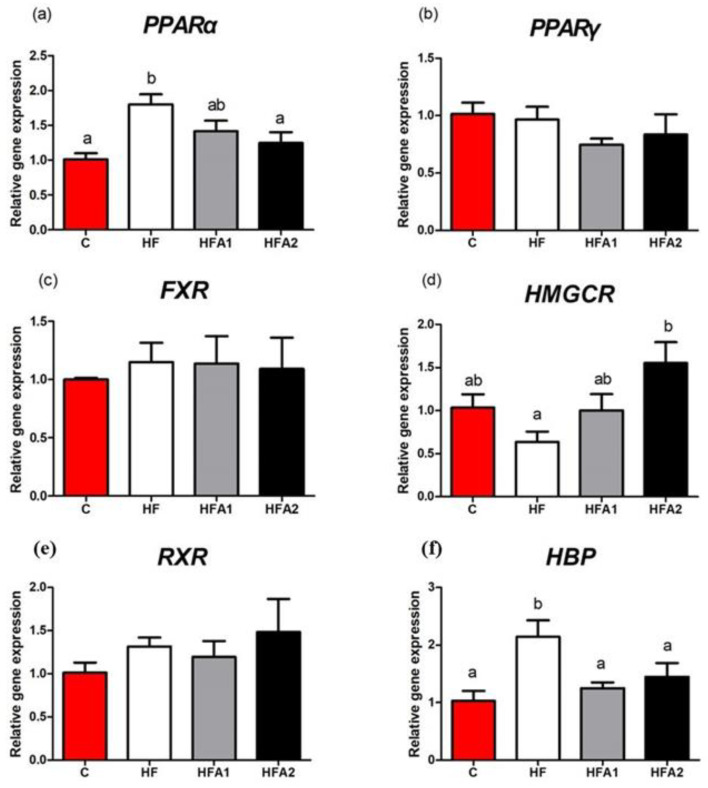
Effects of high-fat diet and astaxanthin on the relative expression of hepatic lipid metabolism-related mRNAs of largemouth bass. (**a**), the mRNA level of *peroxisome proliferator activating receptor α* *(PPARα)* in liver; (**b**), the mRNA level of *peroxisome proliferator activating receptor γ* *(PPARγ) in liver*; (**c**), the mRNA level of *farnesoid X receptor* *(FXR) in liver*; (**d**), the mRNA level of *3-Hydroxy-3-methylglutaryl-CoA Reductase* *(HMGCR)* in liver; (**e**), the mRNA level of *retinoid X receptor α* *(RXRα)*; (**f**), the mRNA level of *lipoprotein binding protein* *(HBP)* in liver. Values were presented as means ± SEM of four replicates, and mean values with unlike letters indicate significant differences (*p* < 0.05).

Figure 2 shows the expression of lipid metabolism-related gene expression. The mRNA level of *peroxisome proliferator activating receptor α* (*PPARα*) and *lipoprotein binding protein* (*HBP*) were significantly higher in fish fed diet HF than those fed diet C (*p* < 0.05). Dietary supplementation with 150 mg kg^−1^ and 75–150 mg kg^−1^ astaxanthin decreased the *PPARα* and *HBP* expression of fish fed HFD, respectively (*p* < 0.05). Dietary supplementation with 150 mg kg^−1^ astaxanthin also down-regulated the *3-hydroxy-3-methylglutaryl-coenzyme A reductase* (*HMGCR*) expression in fish fed HFD (*p* < 0.05). The mRNA levels of *farnesoid X receptor* (*FXR*), *retinoid X receptor* (*RXR*) and *proliferator activating receptor γ* (*PPARγ*) in the liver were similar between fish fed the four diets (*p* > 0.05).

### 2.6. Oxidative Stress and Anti-Oxidative Related Parameters in Liver and Plasma

**Figure 3:**
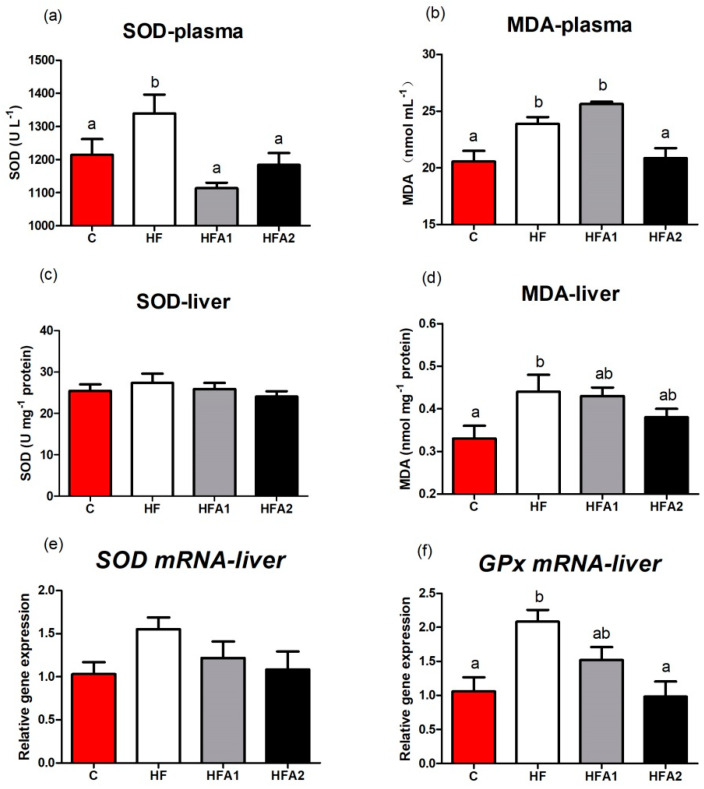
Effects of high-fat diet and astaxanthin on hepatic anti-oxidative ability and oxidative stress of largemouth bass. (**a**), the superoxide dismutase (SOD) activity in plasma; (**b**), the malondialdehyde (MDA) content in plasma; (**c**), the SOD activity in liver; (**d**), the MDA content in plasma; (**e**); the mRNA level of *SOD* in liver; (**f**), the mRNA level of *glutathione peroxidase* (*GSH-px*) in liver. Values were presented as means ± SEM of four replicates, and mean values with unlike letters indicate significantly differences (*p* < 0.05).

The MDA contents in liver and plasma were significantly increased in fish fed diet HF compared with the fish fed control diet (Figure 3), and dietary supplementation with 150 mg kg^−1^ astaxanthin in HFD significantly reduced the MDA content in plasma (*p* < 0.05). Superoxide dismutase (SOD) activity in plasma of fish fed diet HF was significantly higher than fish fed other diets (*p* < 0.05). No difference was observed in SOD activity in the liver among the four treatments (*p* > 0.05). The mRNA level of *GPx* in the liver was higher in fish fed the diet HF than those fed diet C and HFA2 (*p* < 0.05). The expressions of *SOD* in the liver were similar among fish fed the four diets.

### 2.7. Immune Response Related Gene Expression in Liver

**Figure 4:**
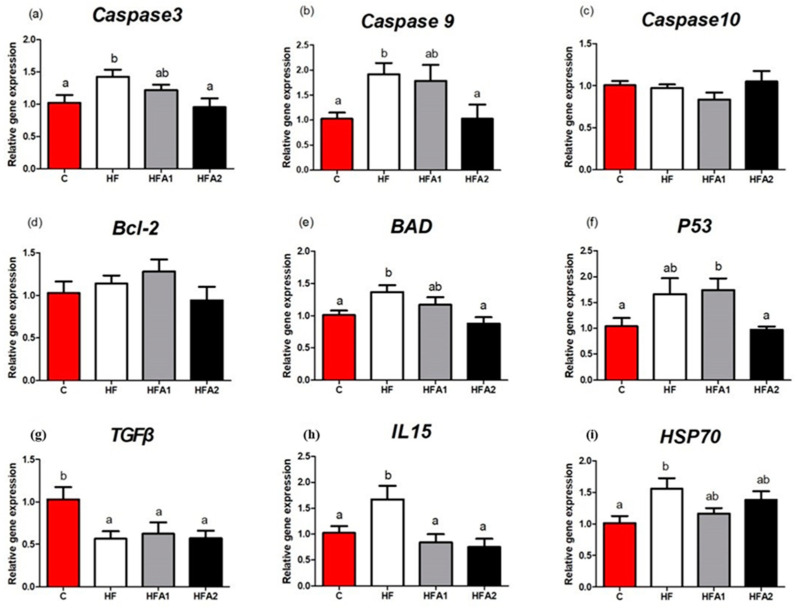
Effects of high-fat diet and astaxanthin on the relative expression of immune response-related mRNAs in the liver of largemouth bass. (**a**), the mRNA level of *cysteine-aspartic proteases-3* *(Caspase3*) in fish fed different diets; (**b**), the mRNA level of *cysteine-aspartic proteases-9* *(Caspase9)* in fish fed different diets; (**c**), the mRNA level of *cysteine-aspartic proteases-10* *(Caspase10)* in fish fed different diets; (**d**), the mRNA level of *B-cell lymphoma-2* *(Bcl-2*) in fish fed different diets; (**e**), the mRNA level of *Bcl-2-associated death protein* *(BAD)* in fish fed different diets; (**f**), the mRNA level of *P53* in fish fed different diets; (**g**), the mRNA level of *transforming growth factor-β* *(TGFβ*) in fish fed different diets; (**h**), the mRNA level of *interleukin 15* *(IL15)* in fish fed different diets; (**i**), the mRNA level of *Heat shock protein 70* *(HSP70)* in fish fed different diets. Values were presented as means ± SEM of four replicates, and mean values with unlike letters indicate significant differences (*p* < 0.05).

The immune response-related gene expressions in the liver of fish fed different diets are shown in Figure 4. The mRNA levels of *Caspase 3*, *Caspase 9*, *Bcl-2-associated death protein* (*BAD*), *IL15* and *heat stress protein 70* (*HSP70*) were significantly higher in the liver of fish fed diet HF compared to those fed the diet C (*p* < 0.05). Dietary supplementation of 150 mg kg^−1^ astaxanthin significantly decreased the mRNA levels of *Caspase 3*, *Caspase 9* and *BAD*. Dietary supplementation of 75–150 mg kg^−1^ astaxanthin also significantly decreased the mRNA levels of *IL15* in the liver. The gene expression of *transforming growth factor β* (*TGFβ*) was significantly higher in fish fed diet C than those fed the other diets (*p* < 0.05). The *P53* mRNA levels were significantly higher in the liver of fish fed diet HFA1 compared to those fed the diets C and HFA2 (*p* < 0.05). There were no differences in the gene expression of *B-cell lymphoma-2* (*Bcl-2*) among the four groups (*p* > 0.05).

## 3. Discussion

HFD has been shown to impair the growth performance of grass carp, giant croaker, tilapia, blunt snout bream and other aquatic animals [27,28,29,30]. In this study, at the end of the feeding trial, WG, survival and SGR were similar in fish fed diet C and HF, which indicated that HFD (18.08%) did not impair the growth performance of largemouth bass. Our previous research indicated 18.08% of dietary lipid impaired the growth of largemouth bass, Zhou et al. (2020) also reported that largemouth bass fed 20% dietary lipid showed lower WG than those fed 10% dietary lipid [13]. Meanwhile, in another study, the optimal lipid requirement of largemouth bass was 18.42% [31]. The different results in lipid requirement of largemouth bass may result from the differences in diet formulation and environmental factors. Present results showed that dietary supplementation of 75 mg kg^−1^ astaxanthin significantly improved the growth performance of fish fed the HFD. Previous studies also reported that dietary supplementation with astaxanthin enhanced the growth of kuruma shrimp, golden pompano and red porgy [22,23,32]. Most of those studies evaluated the effects of astaxanthin supplementation in normal aquatic feeds, this is the first study that reveals the beneficial effects of astaxanthin supplementation on growth performance of fish fed HFD.

HFD usually induces the abnormal accumulation of lipid, and finally causes oxidative stress and inflammation. In this study, we investigated the HFD and astaxanthin on lipid accumulation and lipid metabolism firstly. When largemouth bass fed HFD, higher VSI, HSI and IPF were observed, higher lipid contents in muscle, liver and whole body also were observed. These results indicated HFD induced more lipid accumulation in several tissues (viscera and muscle) of largemouth bass, similar results were found in our previous research [12] and several studies in largemouth bass [31], giant croaker [28] and cobia [10]. The supplementation of astaxanthin in HFD decreased the HSI and IPF of largemouth bass, while having no effects on lipid contents in the whole body, muscle and liver. These results indicate that astaxanthin only reduced the fat deposition of largemouth bass to a certain extent. Similar results were reported in red porgy; dietary supplementation of 100 mg kg^−1^ astaxanthin decreased the lipid contents in the whole body and liver [32]. Meanwhile, 100 mg kg^−1^ dietary astaxanthin had no effects on VSI, HSI and whole-body lipid content of golden pompano [23], and 200–1600 mg kg^−1^ astaxanthin did not affect the whole body lipid content of kuruma shrimp [22]. In the research of mice, Yang et al. (2014) reported that 0.3–3 mg astaxanthin/kg body weight had no effects on body fat of mice [25], Ikeuchi et al. (2007) reported that 6–30 mg astaxanthin/kg body weight decreased the liver weight [33]. These previous research studies indicated that dietary astaxanthin supplementation may have larger effects on liver weight than body fat, which revealed that astaxanthin products may have limited effect to control body fat in humans and other animals.

Although astaxanthin has a limited effect on body lipid deposition, it still affected the lipid metabolism of animals and humans in many previous studies. A meta-analysis of randomized controlled trials revealed that astaxanthin supplementation is associated with an increase in HDL [34]. Similar results were found in the present study; the ratio of HDL/LDL increased when fish fed diet supplemented with 150 mg kg^−1^ astaxanthin. HFD also induced high TG and TC in plasma and liver in this study, astaxanthin supplementation reduced the TG in plasma and TC in the liver. In previous studies, astaxanthin had no effects on TG and TC levels when a normal diet was adopted [34,35]. When mice were fed an HFD, astaxanthin supplementation also showed similar TG and TC lowering effects as in this study [25,26,33]. Jia et al. (2016) reported that astaxanthin modulates the lipid accumulation of high-fat-fed mice via activation of PPARα and inhibition of PPARγ [26]. In this study, the *PPARα* expression in the liver of largemouth bass showed the opposite tendency as in mice. PPARα is essential in the regulation of genes encoding fatty acid transport, metabolism and fatty acid oxidation in the liver [36]. The lower expression of *PPARα* in the HFA2 group compared to the HF group indicated the lower lipid content in the liver, which was positively correlated with the HSI results. *HMGCR* is the rate-limiting enzyme for cholesterol synthesis of largemouth bass [37], the low *HMGCR* expression may associate with the high hepatic TC content. In this study, dietary astaxanthin supplementation enhanced the hepatic mRNA expression of *HMGCR*, similar results were found in mice [33]. In this study, the mRNA expression of lipid metabolism-related gene expressions was consistent with the results of plasma lipids and body fat, which clearly showed that the gene revealed that dietary astaxanthin supplementation may affect the hepatic fatty acid oxidation and cholesterol synthesis of high-fat-fed largemouth bass.

Some previous studies suggested HFD inducing ROS production, inflammatory response, and apoptosis in fish and mammals [31,38,39,40]. Excessive production of ROS will cause damage to the organism, and antioxidant enzymes such as SOD and GPx are the main participators for eliminating ROS [41]. As a powerful antioxidant, astaxanthin also has been shown to enhance the anti-oxidative ability of animals under stress or pathological state [19,21,42,43]. In this study, SOD activity in plasma and *GPx* mRNA level in the liver were increased in fish fed HF diet to eliminate the excessive ROS, and dietary supplementation of astaxanthin restored the hepatic redox state of largemouth bass. Excessive production of ROS usually leading to oxidative stress, MDA, a product of lipid peroxidation, is considered a critical marker of oxidative stress [44]. In this study, MDA contents in plasma and liver were higher in high-fat-fed fish, which revealed that HFD induced oxidative stress in largemouth bass. Dietary supplementation of 150 mg kg^−1^ astaxanthin reduced the MDA content in plasma of largemouth bass, which indicated that suitable astaxanthin alleviated the oxidative stress brought by HFD. The gene expression and activity of anti-oxidative enzymes and MDA contents clearly indicated the antioxidant system of largemouth bass was damaged by HFD, and the damage was alleviated by astaxanthin supplementation. Similar results were reported by Yang et al. (2014) [25], the anti-oxidative ability of mice fed HFD was enhanced by astaxanthin supplementation. Bhuvaneswari et al. (2014) also reported astaxanthin supplementation decreased the ROS induced by HFD [45].

Besides the anti-oxidative ability, the immune response of fish also was affected by HFD and astaxanthin. IL 15 and TGF-β are two critical inflammatory cytokines [12], HFD increased the mRNA expression of *IL15* and decreased the mRNA expression of *TGF-β* in this study, which indicated hepatic inflammation of largemouth bass may be induced by HFD. Dietary supplementation of astaxanthin reduced the expression of *IL15*, but could not enhance the expression of anti-inflammatory cytokines *TGF-β*. In previous studies, astaxanthin supplementation reduced the TNFα and IL6 of mice fed HFD [25,26], astaxanthin also modulating the immune response of animals through regulating the expression of cytokines. Apoptosis is a necessary condition for the development and homeostasis of cells [46]. In response to a death signal, activation of the intrinsic apoptotic pathway results in altered expression of the genes including anti-apoptotic (*Bcl-2*, *Bcl-xl* and *BAG*) and pro-apoptotic (*Bax*, and *BAD*) [46,47,48]. The caspase family is also critical in mediating apoptosis, with Caspase 3 being the key executive molecule, and Caspase 9 being the upstream signaling of Caspase 3 [49,50]. Activated caspase triggers compensatory proliferation, referred to as apoptosis-induced proliferation which maintains tissue homeostasis following massive stress-induced cell death, regenerating damaged structures [51]. In the present study, gene expression of *BAD*, *Caspase 3* and *Caspase 9* in the liver of largemouth bass fed HFD were significantly elevated, suggesting HFD may induce apoptosis of largemouth bass. Similar results had been reported in blunt snout bream fed HFD [30]. The high expression of *Caspase3*, *Caspase 9* and *BAD* were reduced by the supplementation of 150 mg kg^−1^ astaxanthin, which indicating that HFD-induced apoptosis was alleviated by astaxanthin. These results are consistent with research reported in mice that dietary astaxanthin supplementation could inhibit apoptosis induced by HFD [45]. Although protein expression samples failed to further verify the apoptosis, the present results from gene expression is still robust enough to suggest that astaxanthin supplementation may inhibit apoptosis in largemouth bass by the down-regulation of pro-apoptotic genes.

Overall, our results firstly proved that astaxanthin supplementation decreased the liver weight and alleviated the oxidative stress and inflammation of fish fed HFD, which was similar to many studies in mice. These results revealed the therapeutic potential of astaxanthin to liver inflammation induced by HFD in humans and other animals.

## 4. Materials and Methods

All the experiments were conducted according to the recommendations in the Guide for the Care and Use of Laboratory Animals of the National Institutes of Health. The study protocol and all experimental procedures were approved by the Experimental Animal Ethics Committee of Sun Yat-Sen University (EAEC201809122).

### 4.1. Diet Preparation

Four isonitrogenous diets were formulated (Table 4), a control diet (10.87% lipid, C), a high-fat diet (18.08% lipid, HF), and the HF diet supplemented with 75 and 150 mg kg^−1^ astaxanthin (HFA1 and HFA2, respectively). The synthetic astaxanthin (Carophyll Pink, 10%) was obtained from DSM (Heerlen, Netherlands). The lipid levels were adopted according to our previous study [12]. The ingredients were smashed and well mixed, the 2.5 mm diameter pellets were extruded using a twin-screw cooking extruder (HQ, Zhaoqing, China). The diets were air-dried to approximately 10% moisture and stored at −20 °C until used.

### 4.2. Fish Rearing and Experimental Conditions

Largemouth bass were provided by a local hatchery (Foshan, China). Fish were firstly acclimated in an indoor recirculating aquaculture system for two weeks. Before the experiment, 480 fish of similar size (15.25 ± 0.02 g) were randomly allocated to 16 fiberglass tanks (300 L), each tank with 30 fish. Each diet was distributed to four tanks, fish were fed twice (8:30 and 16:30) daily to visual satiation for 8 weeks. During the experiment, the water temperature was 26.6 ± 1.3 °C and pH was 7.74 ± 0.06. Total ammonia nitrogen was 0.12 ± 0.02 mg L^−1^, and dissolved oxygen was 8.23 ± 0.33 mg L^−1^. Natural light–dark cycle was employed during the experiment.

### 4.3. Sample Collection and Biochemical Composition Analysis

Fish fasted for 24 h after the last meal, then were counted and weighted. Twelve fish per tank were randomly selected and anesthetized in MS-222 (Sigma, Santa Clara, CA, USA, 10 mg L^−1^) for sampling. Two fish per tank were randomly selected as the whole-body samples. Six fish per tank were collected blood from caudal vein with heparinized syringes, and weighed the whole fish, viscera and liver. Blood was freeze centrifuged at 3000× *g* for 10 min (Centrifuge MR23i, Jouan, Paris, France), then supernatant was collected, immediately frozen and stored at −80 °C before analysis. Liver samples were rapidly frozen in liquid nitrogen and stored at −80 °C. The whole body and muscle samples were stored at −20 °C. Livers from two fish per tank were collected for oil red O staining.

### 4.4. Proximate Analysis of Diets and Body Composition

Moisture, crude protein, crude lipid, and ash contents in the diets, muscle and whole body were determined by standard methods [52]. Moisture was determined by oven drying at 105 °C until a constant weight was obtained. Crude protein content (N × 6.25) was determined according to the Kjeldahl method and using an Auto Kjeldahl System (1030-Autoanalyzer; Tecator, Helsingborg, Sweden). Crude lipid was exacted and determined by Soxtec extraction System HT (Soxtec System HT6, Tecator). Ash content was determined at 550 °C for 6 h in a muffle furnace (M110, Thermo Scientific, Waltham, MA, USA).

### 4.5. Antioxidative Related Parameters Analysis and Biochemical Index Assays

Liver samples were homogenized in ice-cold saline (8.6 g L^−1^ NaCl in ddH_2_O, 1:9 dilution), then freeze centrifuged at 4000 rpm for 10 min, the supernatants were collected and stored at −80 °C until analyzed. The SOD activity and the contents of MDA, TG, TC, HDL-C and LDL-C were measured by commercial kits (Nanjing Jiancheng Bioengineering Institute, Nanjing, China). MDA was measured by thiobarbituric acid method [53]. SOD was measured by hydroxylamine method [54]. TG and TC contents were determined by enzymatic hydrolysis method [55] and cholesterol oxidase–peroxidase method [56]. LDL-C and HDL-C contents were measured by the method described by Liu et al. (2002) [57]. Protein concentrations of the hepatic suspension were measured by folin phenol method [58].

### 4.6. Quantitative Real-Time PCR Analysis

Quantitative real-time PCR analysis method was described in a previous study [38]. Real-time PCR was performed using SYBR^®^ Premix Ex TaqTM II (Takara, Japan) and quantified on the LightCycler 480 (Roche Applied Science, Basel, Switzerland). An amount of 400 nM of forward and reverse specific primers, 10 ng of cDNA template and nuclease-free water to a final volume of 10 μL, then using the following program: denaturation step at 95 °C for 1 min, followed by 40 amplification cycles of 5 s denaturation at 95 °C, 15 s annealing at 60 °C and 20 s extension at 72 °C, followed by a melt-curve analysis and cooling to 4 °C. The efficiency of primers was evaluated by serial dilutions and all of them were close to 100% (Table 5).

Relative expression levels of target genes were calculated based on the equation of 2^−ΔΔCT^ [59], and elongation factor 1α (EF1α) and β-actin were used as the reference genes. Three technical replicates were conducted for each sample.

**Table 5:** Real-time PCR primer sequences.

Gene	Primer Sequence	Products	Sources
EF1α F	GGCTGGTATCTCCAAGAACG	239	[60]
EF1α R	GTCTCCAGCATGTTGTCWCC		
GSH-px F	GGGGCTCCACCTGCTTCTTG	/	FJ030930
GSH-px R	ACCCCTCTGCTCAGGCATTT		
SOD F	TGGCAAGAACAAGAACCACA	167	FJ030929
SOD R	CCTCTGATTTCTCCTGTCACC		
FXR F	AGAAATGGCAACAAGTCAA	77	KT827789.1
FXR R	CACGGTCCAGAGAGAGAAA		
PPARα F	CCACCGCAATGGTCGATATG	161	[61]
PPARα R	TGCTGTTGATGGACTGGGAAA		
HBP F	AAATCCAAATCCCACGAC	134	KF652241.1
HBP R	CACCCTCTCTACAGCACG		
PPAR-γ F	CCTGTGAGGGCTGTAAGGGTTT	118	[61]
PPAR-γ R	TTGTTGCGGGACTTCTTGTGA		
IL-15 F	GTATGCTGCTTCTGTGCCTGG	82	[61]
IL-15 R	AGCGTCAGATTTCTCAATGGTGT		
TGF-β F	GCTCAAAGAGAGCGAGGATG	118	[61]
TGF-β R	TCCTCTACCATTCGCAATCC		
Caspase3 F	GCTTCATTCGTCTGTGTTC	98	[61]
Caspase3 R	CGAAAAAGTGATGTGAGGTA		
Caspase9 F	CTGGAATGCCTTCAGGAGACGGG	125	[61]
Caspase9 R	GGGAGGGGCAAGACAACAGGGTG		
Caspase10 F	CAAACCACTCACAGCGTCTACAT	146	[61]
Caspase10 R	TGGTTGGTTGAGGACAGAGAGGG		
HSP 70 F	CAGTGATGAAGACAAGCAGAAGA	163	[62]
HSP 70 R	GCCACCAGCACTCTGATACA		
Bcl-2 F	CCATCCACGACGAACCTG	75	/
Bcl-2 R	GGCGTATCGCTGCTCAAACT		
Bcl-xL F	CATCCTCCTTGGCTCTGG	141	/
Bcl-xL R	GGGTCTGTTTGCCTTTGG		
RXRα F	AGCAGAGCAGGCAGTGGA	144	KT827793.1
RXRα R	CGTTGGGCGAGTTGGAT		
HMGCR F	GGTGGAGTGCTTAGTAATCGG	125	/
HMGCR R	ACGCAGGGAAGAAAGTCAT		
BAD F	CACATTTCGGATGCCACTAT	116	/
BAD R	TTCTGCTCTTCTGCGATTGA		
P53 F	AGATTGAATGGTGGTGGG	144	/
P53 R	GTTCTGGCGGACTGGA		

EF1α, elongation factor 1α; GSH-px, glutathione peroxidase; SOD, superoxide dismutase; FXR, farnesoid X receptor; Caspase 3, cysteine-aspartic proteases-3; Caspase 9, cysteine-aspartic proteases-9; Caspase 10, cysteine-aspartic proteases-10; IL 15, interleukin 15; TGFβ, transforming growth factor-β; Bcl-2, B-cell lymphoma-2; Bcl-xl, B-cell lymphoma-xl; RXRα, retinoid X receptor α; HMGCR, 3-Hydroxy-3-methylglutaryl-CoA Reductase; Bad, Bcl-2 associated death protein. PPARα, peroxisome proliferator activating receptor α; PPAR-γ, peroxisome proliferator activating receptor γ; HBP, high density lipoprotein binding protein; HSP 70, heat stress protein 70.

### 4.7. Oil Red O Staining

Oil red O staining was performed to determine the number of neutral lipids deposited in the liver, the method was described as Yin et al. (2021) [12]. Liver samples were firstly fixed with 4% paraformaldehyde and then frozen with liquid nitrogen. Secondly, the sectioned slides (6–10 μm thickness) were stained with oil red O solution (3 g L^−1^, Servicebio, Wuhan, China) for 8–10 min. Then, the nuclei were counterstained with hematoxylin solution (Servicebio, Wuhan, China) for 3–5 min. After staining, the slides were washed and a cover glass was fixed to the slide with glycerol jelly mounting medium (Servicebio, Wuhan, China). The lipid droplets were observed using a light microscope (Nikon Eclipse Ni-U, Tokyo, Japan) at 100× magnification.

### 4.8. Calculations and Statistical Analysis

All data are presented as means ± SEM and analyzed by one-way ANOVA using SPSS 19.0 (SPSS). All data were checked for normality and homogeneity of variance before analysis. When there were significant differences (*p* < 0.05), the group means were further compared using Duncan’s multiple range test. When data did not have a homogeneous variation, the non-parameter Kruskal–Wallis test was applied, and followed by all pairwise multiple comparisons if the results showed a significant difference (*p* < 0.05).

## 5. Conclusions

In conclusion, the present study firstly indicated that dietary astaxanthin enhanced the growth performance of largemouth bass fed HFD. The supplementation of astaxanthin improved the lipid metabolism of largemouth bass fed HFD, as well as reduced the oxidative stress and apoptosis induced by HFD in fish. The suitable supplemental level of astaxanthin based on the growth of largemouth bass fed HFD is 75 mg kg^−1^, while based on anti-oxidative ability and immune response largemouth bass fed HFD is 150 mg kg^−1^.

## Figures and Tables

**Table 1:** Effects of dietary supplementation of astaxanthin on growth performance, feed utilization and somatic parameters of juvenile largemouth bass fed high-fat diets (HFDs).

	C	HF	HFA1	HFA2
IBW (g)	15.26 ± 0.02	15.26 ± 0.01	15.25 ± 0.02	15.23 ± 0.03
FBW (g)	64.45 ± 1.21 a,b	62.24 ± 1.17 a	67.04 ± 1.34 b	64.91 ± 1.13 a,b
WG (%)	322.88 ± 8.05 a,b	307.92 ± 7.43 a	339.53 ± 8.26 b	326.34 ± 8.42 a,b
SR (%)	96.67 ± 1.67	96.25 ± 2.39	96.25 ± 3.75	95 ± 2.89
SGR (% day^−1^)	2.57 ± 0.04 ab	2.51 ± 0.03 a	2.64 ± 0.03 b	2.56 ± 0.04 a,b
PER	2.08 ± 0.03 a	2.19 ± 0.06 a	2.36 ± 0.03 b	2.38 ± 0.04 b
CF (g cm^−3^)	2.31 ± 0.04	2.3 ± 0.05	2.27 ± 0.03	2.29 ± 0.06
VSI (%)	8.06 ± 0.16 a	8.98 ± 0.2 b	9.12 ± 0.18 b	8.89 ± 0.17 b
HSI (%)	2.3 ± 0.06 a	2.53 ± 0.06 b	2.29 ± 0.07 a	2.1 ± 0.09 a
IPF (%)	1.31 ± 0.13 a	2.09 ± 0.09 c	1.88 ± 0.16 b,c	1.58 ± 0.18 a,b

Initial body weight (IBW); Final body weight (FBW, g) = final body weight/final number of fish; Weight gain rate (WG, %) = 100 × (final body weight − initial body weight)/initial body weight; Survival rate (SR,%) = 100 × (final number of fish)/(initial number of fish); Specific growth rate (SGR, % day^−1^) = 100 × (Ln final individual weight − Ln initial individual weight)/number of feeding days; Protein efficiency ratio (PER) = total weight gain(g)/protein intake (g); Condition factor (CF, g cm^−3^) = 100 × (body weight, g)/(body length, cm)^3^; Viscerosomatic index (VSI, %) = 100 × (viscera weight, g)/(whole bodyweight, g); Hepatosomatic index (HSI, %) = 100 × (liver weight, g)/(whole body weight, g); Intraperitoneal fat ratio (IPF) = 100 × (intraperitoneal fat weight/whole body weight). Results were presented as “mean ± SEM” of four replicates, and mean values on the same line with different letters indicate significantly different (*p* < 0.05), while with the same letter or no letter mean no significant difference (*p >* 0.05).

**Table 2:** Effects of dietary astaxanthin supplementation on whole-body and muscle proximate composition of largemouth bass fed HFD (wet weight %).

	C	HF	HFA1	HFA2
Whole-body				
Moisture	70.38 ± 0.22 b	69.24 ± 0.11 a	69.01 ± 0.19 a	68.65 ± 0.26 a
Crude Protein	17.30 ± 0.15	16.97 ± 0.12	17.10 ± 0.16	17.32± 0.15
Crude Lipid	7.32 ± 0.81 a	9.20± 0.21 b	9.08 ± 0.66 b	9.64 ± 0.31 b
Ash	3.87 ± 0.11 b	3.74 ± 0.08 a,b	3.69 ± 0.05 a,b	3.61 ± 0.05 a
Muscle				
Moisture	78.01 ± 0.18	77.92 ± 0.11	77.72 ± 0.09	77.66 ± 0.10
Crude Protein	19.90 ± 0.19	19.73 ± 0.17	19.92 ± 0.06	19.76 ± 0.23
Crude Lipid	0.76 ± 0.11 a	1.46 ± 0.19 b	1.30 ± 0.05 b	1.40 ± 0.15 b
Ash	2.87 ± 0.04 b	2.68 ± 0.06 a	2.65 ± 0.06 a	2.57 ± 0.03 a

Results were presented as “mean ± SEM” of four replicates, and mean values on the same line with different letters indicate significantly different (*p* < 0.05), while with the same letter or no letter mean no significant difference (*p* > 0.05).

**Table 3:** Effects of dietary astaxanthin supplementation on Biochemical parameters of plasma and liver in juvenile largemouth bass fed high-fat diets.

	C	HF	HFA1	HFA2
Plasma	-	-	-	-
TG (mmol L^−1^)	5.1 ± 0.43 a	8.51 ± 1.07 b	5.62± 0.38 a	4.57± 0.82 a
TC (mmol L^−1^)	15.63 ± 0.95 a	19.68 ± 0.58 b	16.17 ± 0.37 a,b	16.45 ± 0.99 a,b
HDL/LDL	0.32 ± 0.02 a,b	0.24 ± 0.02 a	0.27 ± 0.03 a	0.37 ± 0.03 b
LDH (U L^−1^)	615.37 ± 14.07	681.88 ± 41.96	626.67 ± 19.97	628.24 ± 23
Liver	-	-	-	-
Lipase (U g prot^−1^)	0.17 ± 0.01	0.16 ± 0.01	0.16 ± 0.02	0.20 ± 0.02
TG (mmol g prot^−1^)	0.06 ± 0.00 a	0.07 ± 0.01 a,b	0.08 ± 0.01 b	0.07 ± 0.0 a,b
TC (mmol g prot^−1^)	0.04 ± 0.01 a	0.08 ± 0.01 b	0.05 ± 0.01 a	0.05 ± 0.01 a,b

TG, total triglyceride; TC, total cholesterol; HDL-C, high-density lipoprotein; LDL-C, low-density lipoprotein; LDH, lactate dehydrogenase; Lipase. Results were presented as “mean ± SEM” of four replicates, and mean values on the same line with different letters indicate significantly different (*p* < 0.05), while with the same letter or no letter mean no significant difference (*p* > 0.05).

**Table 4:** Formulation and proximate composition of the experimental diets (% dry matter).

Ingredients (%)	C	HF	HFA1	HFA2
Fish meal	50	50	50	50
Wheat flour	10.9	10.9	10.825	10.75
Chicken meal	6.00	6.00	6.00	6.00
Microcrystalline cellulose	7	0	0	0
Beer yeast	3	3	3	3
Shrimp meal	3	3	3	3
Soy protein concentrate	1	1	1	1
Soybean oil	2	9	9	9
Fish oil	3.00	3.00	3.00	3.00
Choline chloride (50%)	0.5	0.5	0.5	0.5
Monocalcium phosphate	1.50	1.50	1.50	1.50
Vitamin mixture ^a^	1	1	1	1
Mineral mixture ^b^	1	1	1	1
Vc phosphonate	0.1	0.1	0.1	0.1
Astaxanthin ^c^	0.00	0.00	0.075	0.15
Soybean protein concentrate	10	10	10	10
Proximate composition (%)	-	-	-	-
Moisture	6.60	6.54	6.34	5.68
Crude protein	49.07	48.48	49.07	49.18
Crude lipid	10.87	18.08	18.06	18.21
Ash	15.59	15.40	15.62	16.84

^a^ Vitamin premix (IU or mg kg^−1^ diet): vitamin B1, 800; vitamin B2, 1600; vitamin B6, 1200; nicotinic acid, 3800; D-Ca pantothenate, 2500; myo-inositol, 4000; d-biotin, 40; folic acid, 320; vitamin A, 400,000 IU; vitamin E, 16,000; vitamin K3, 600; vitamin D3, 80,000 IU; vitamin B12, 4; vitamin C, 35,000. ^b^ Mineral premix (mg kg^−1^ diet): magnesium, 3000; iron, 1800; manganese, 800; iodine, 250; copper, 350; zinc, 6500; selenium, 15; cobalt, 60. ^c^ The synthetic astaxanthin (Carophyll Pink, 10%) was obtained from DSM (The Netherlands).

## Abbreviations

*BAD*	*Bcl-2-associated death protein*
*Bcl-2*	*B-cell lymphoma-2*
*Caspase3*	*cysteine-aspartic proteases-3*
*Caspase9*	*cysteine-aspartic proteases-9*
*Caspase10*	*cysteine-aspartic proteases-10*
CF	condition factor
EF1α	elongation factor 1α
FBW	final body weight
*FXR*	*farnesoid X receptor*
HDL-C	high-density lipoprotein cholesterol
HFD	high fat diet
HIS	hepatosomatic index
*HBP*	*lipoprotein binding protein*
*HMGCR*	*3-hydroxy-3-methylglutaryl-coenzyme A reductase*
*HSP70*	*Heat shock protein 70*
IBW	initial body weight
IPF	intraperitoneal fat ratio
LDL-C	low-density lipoprotein cholesterol
*IL15*	*interleukin 15*
MDA	malondialdehyde
PER	protein efficiency ratio
*PPARα*	*peroxisome proliferator activating receptor α*
*PPARγ*	*proliferator activating receptor γ*
*RXR*	*retinoid X receptor*
SOD	superoxide dismutase
TC	total cholesterol
TG	total triglyceride
*TGFβ*	*transforming growth factor-β*
VSI	viscerosomatic index
WG	weight gain
